# Determination of Antimony (III) in Real Samples by Anodic Stripping Voltammetry Using a Mercury Film Screen-Printed Electrode

**DOI:** 10.3390/s90100219

**Published:** 2009-01-08

**Authors:** Olga Domínguez-Renedo, M. Jesús Gómez González, M. Julia Arcos-Martínez

**Affiliations:** Departamento de Química, Área de Química Analítica, Facultad de Ciencias, Universidad de Burgos, Plaza Misael Bañuelos s/n, E-09001 Burgos, Spain; E-Mails: olgado@ubu.es; mjgomez@ubu.es

**Keywords:** antimony, anodic stripping voltammetry, mercury film electrode, screen-printed

## Abstract

This paper describes a procedure for the determination of antimony (III) by differential pulse anodic stripping voltammetry using a mercury film screen-printed electrode as the working electrode. The procedure has been optimized using experimental design methodology. Under these conditions, in terms of Residual Standard Deviation (RSD), the repeatability (3.81 %) and the reproducibility (5.07 %) of the constructed electrodes were both analyzed. The detection limit for Sb (III) was calculated at a value of 1.27×10^−8^ M. The linear range obtained was between 0.99 × 10^−8^ − 8.26 × 10^−8^ M. An analysis of possible effects due to the presence of foreign ions in the solution was performed and the procedure was successfully applied to the determination of antimony levels in pharmaceutical preparations and sea water samples.

## 1. Introduction

Heavy metals belong to a class of pollutant that can produce undesirable effects, even though they might be present in minuscule quantities [1]. They are extremely toxic, non-biodegradable and tend to bio-accumulate in animal and vegetable tissues [2]. Antimony and its compounds are listed as pollutants of priority interest by the Environmental Protection Agency of the United States and the Council of the European Communities [3]. Antimony may be found in the environment because of various anthropogenic activities. Antimony-containing compounds are used in the manufacture of glass and ceramics as well as in fire retardants. Road traffic is also a significant source, as it is used in brake linings and tire vulcanization processes that require Sb-containing additives [4]. Furthermore, ultra-trace level concentrations of antimony are commonly found in environmental materials such as seawater and marine organisms [5-7]. In the other hand, water bottled commercially in polypropylene (PP), often suffers from antimony contamination from the containers [8].

Environmental measurements of antimony levels are normally carried out using conventional analytical techniques such as atomic absorption spectroscopy [9], inductively coupled plasma-atomic emission spectroscopy (ICP-AES) and inductively coupled plasma-mass spectrometry (ICP-MS). These techniques are impractical for on-site screening or for quantification as part of a decision tool owing to their size and high labor and analytical costs. Hence, there is a need for portable analytical systems that can be met using electrochemical methods [10]. Electroanalytical techniques bring with them important advantages such as, high sensitivity, low detection limits, relative simplicity, low costs and portable field-based equipment able to determine trace elements. For this reason, electrochemical techniques offer an interesting alternative to methods that are currently in use. Voltammetric methods are among the electrochemical techniques described for the analysis of antimony. These are relatively widespread and, due to their accuracy and sensitivity, have contributed greatly to its determination at trace level [11]. Adsorptive stripping voltammetry has also been used in the determination of antimony following adsorptive accumulation of antimony complexes using chloranilic acid as a complexing agent [12, 13].

Screen-printed electrodes (SPEs) are planar devices with plastic substrates that are coated with layers of electroconductive and insulating inks at controlled thickness. The advent of screen-printed (thick-film) technology has made it possible to mass-produce inexpensive disposable electrodes for use with electrochemical instruments [14-19]. Their use in potentiometric, amperometric and voltammetric devices have been reported for the detection of heavy metals such us copper [2, 20-23], lead [9, 21, 24-27], cadmium [2, 9, 21, 25, 27] and mercury [22] although they have not been frequently used in the determination of antimony.

The aim of this work is to determine antimony (III) by means of a new differential pulse anodic stripping voltammetry (DPASV) method using SPEs. In order to do this, a three-electrode configuration (a graphite-working electrode, a silver-counter electrode and a silver/silver chloride-reference electrode) was produced using screen-printing technology. The graphite surface was modified by the deposition of a mercury film.

## 2. Methods

### 2.1. Optimization of Experimental Variables

**Figure 1.**
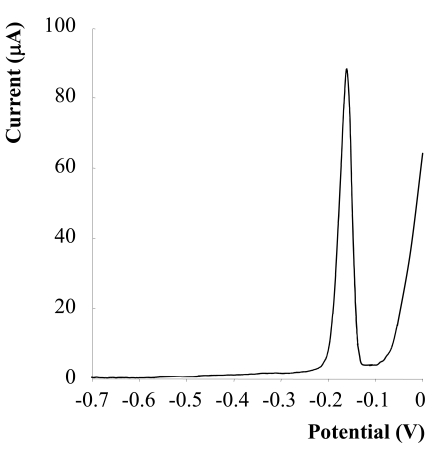
Differential Pulse Anodic Stripping Voltammogram of 10^−6^ M Sb (III) in 3.00 M HCl, E_dep_ = −0.70 V and t_dep_ = 718 s, using a mercury film modified graphite screen-printed electrode (Mercury film: C_Hg_ = 800 mg/l, E_dep_ = −0.90 V and t_dep_ = 600 s).

It is well known that the voltammetric determination of Sb(III) using carbon screen printed working electrodes is not suitable, however, a modification of the graphite electrode by the deposition of a mercury film can produced signals of a high quality. In Figure 1 is possible to observe the analytical signal obtained for Sb(III) using SPCEs modified with mercury.

The intensity of the observed electrochemical response is influenced by various factors related to the DPASV technique used and by the characteristics of the mercury coating (thickness, resistance), which in turn are conditioned by a number of experimental parameters that affect its formation. For this reason, it is necessary to optimize all experimental parameters, which may have an influence on the electrochemical response in order to ensure the quality of the results. The response to be optimized was the intensity (Ip) of the electrochemical peak obtained for a sample containing a concentration of Sb (III) of 1.00 × 10^−6^ M (Figure 1).

In the formation of the mercury film, the factors to be optimized were the concentration of the Hg [28] solution and time and deposition potential of the mercury film [t_dep_ (Hg film)]. In the determination of Sb (III) by anodic stripping voltammetry, the factors to be optimized were the acidity and features of the supporting electrolyte and the potential and time of deposition (E_dep_, t_dep_).

Taking into account previous experiences described for the determination of Sb (III) [29, 30] using mercury film glassy carbon electrodes, an accumulation potential of −0.90 V was chosen for the formation of the mercury film. In effect, it can be experimentally proved that for potentials more positive than −0.90 V, no deposition of the mercury film was observed and for potentials lower than −0.990 V poor results were obtained.

The concentration of the mercury solution was fixed at 800 mg L^−1^. For lower mercury concentrations of mercury no mercury film deposition was observed.

The use of 3.00 M HCl as the supporting electrolyte in the DPASV determination of Sb(III) gave rise to high and well-defined oxidation peaks [30].

For optimization of the three remaining factors, experimental design methodology was applied. Both 2^n^ (n = number of variables) factorial design and 2^K^ (k = number of variables) central composite designs were applied, with replication in the central point in order to estimate the experimental error. A high and a low level were selected for each of the experimental design factors to be optimized. The next step consisted of experiments using all the possible combinations.

The first phase in the optimization process involved a 2^3^ factorial design. The values of the high (+) and low (-) levels and the central point [31] for each factor were as follows:
t_dep_ (Hg film) (+) = 600 st_dep_ (Hg film) (0) = 390 st_dep_ (Hg film) (-) = 180 st_dep_ (+) = 600 st_dep_ (0) = 390 st_dep_ (-) = 180 sE_dep_ (+) = −0.40 VE_dep_ (0) = −0.60 VE_dep_ (-) = −0.80 V

**Figure 2.**
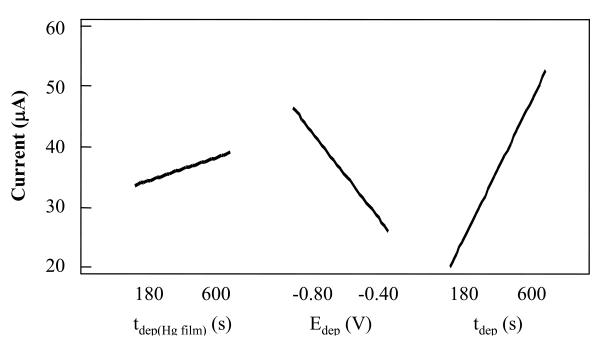
Influence of the main factors in the response in the 2^3^ factorial design for optimization of experimental variables in Sb (III) determination by DPASV.

From analysis of the variance (ANOVA) in Table 1, it may be deduced that t_dep_ (Hg film) is not a significant factor and can be fixed, although the others are significant factors. Analysis of the principal factors, set out in Figure 2, indicates that the signal is improved when higher values are assigned to the t_dep_ and when more negative accumulation potentials are used. As already mentioned, t_dep_ (Hg film) is not a significant factor, nevertheless, it may be seen in Figure 2 that the signal is slightly higher when working with higher times. For that reason, a t_dep_ (Hg film) = 600 s was selected for subsequent experiments.

Mindful of previous observations, the second phase involved a 2^2^ central composite design. The new high (+), low (-) and central [29] levels for each factor were:
t_dep_ (+) = 900 st_dep_ [29] (0) = 600 st_dep_ (-) = 300 sE_dep_ (+) = −0.40 VE_dep_ [29] (0) = −0.80 VE_dep_ (-) = −1.20 V

**Figure 3.**
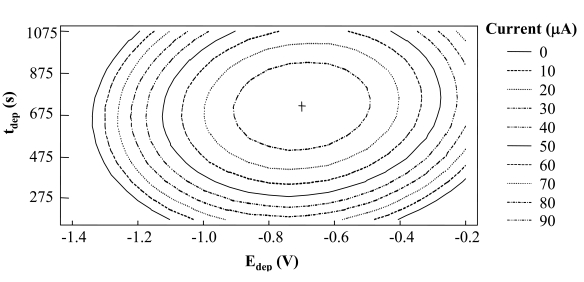
Level curves for the 2^2^ central composite design for optimization of experimental variables in Sb (III) determination by DPASV.

From the analysis of these results (Table 2), it may be deduced that both parameters are significant factors. Nevertheless, a point of maximum intensity may also be observed in Figure 3 that corresponds to an accumulation potential of −0.70 V and an accumulation time of 718 s.

On the basis of these results, the optimum values of the experimental variables for the determination of Sb (III) by anodic stripping voltammetry are as follows:

Mercury film: C_Hg_ = 800 mg L^−1^, E_dep_ = −0.90 V and t_dep_ = 600 s
Determination of antimony: HCl 3.00 M, E_dep_ = −0.70 V and t_dep_ = 718 s

This design led to a 21-fold improvement in the peak current, i_P_, and to more easily quantifiable signals.

## 3. Results and Discussion

Calibration was performed using least-median-squares regression (LMS) to detect the existence of anomalous points [32], which might have led to incorrect adjustments altering the sensitivity and the detection limit. The criterion is to minimize the median of squares of the differences between the experimental and the calculated values. LMS regression has the advantage of being able to detect anomalous points regardless of whether they are “outliers” or “leverage” points, seeking a linear range in which at least 50% of the data are aligned.

The strategy followed consisted of two steps. In the first, the LMS regression was used to detect anomalous points, taking “outliers” to be points where the absolute value of the standardized residual was greater than 2.50 and “leverage” points as those where the absolute value of their resistant diagnostic was greater than 2.50. When both of these parameters were above 2.50, the point was considered as an “outlier-leverage”. In the second step, the anomalous points detected in this way were eliminated and a regression based on the ordinary least squares (OLS) criterion was carried out, to obtain optimal precision and accuracy of both slope and intercept.

The calibration equation obtained by DPASV for standard solutions containing Sb (III) concentrations of between 0.99 × 10^−8^ and 8.26 × 10^−8^ M was:

I = 1.25 ± 0.13 + (0.36 ± 0.03) × 10^8^ C
  (Number of experimental points n = 8; R^2^ = 0.99 and Standard deviation (S_yx_) = 0.08)

A key feature of an analytical method is the detection limit, the smallest concentration of the analyte that can be detected to a specified degree of certainty. The calculation of the detection limit, based on the variability of ten samples with a very low analyte concentration, was calculated according to [33] and ISO 11843-2 [34]. At the chosen probability level of 5% (α = β = 0.05), the detection limit was 1.27 × 10^−8^ M.

### 3.1. Repeatability and Reproducibility

The repeatability of the procedure was analyzed by making successive measurements with the same sensor. Three calibration lines were constructed for Sb (III) concentrations ranging from 2.97 × 10^−7^ M to 10.09 × 10^−7^ M. Having eliminated the anomalous points, the calibration parameters shown in Table 3 were obtained. The standard deviation (RSD) associated with the slopes of these calibrations curves was 3.81 %.

The reproducibility of the method was also checked. The slopes of the five calibration lines (having eliminated the anomalous points), constructed with different sensors were analyzed. The calibration curves were constructed for Sb (III) concentrations ranging from 2.97 × 10^−7^ M to 10.09 × 10^−7^ M. The residual standard deviation (RSD) associated with the slopes of these calibrations curves was 5.21%.

### 3.2. Interferences

An analysis of any possible effects caused by the presence of foreign ions produced the following results. From among all the metallic ions analyzed – As (III), As (V), Cd (II), Cu (II), Fe (II), Fe (III), Ni (II), Pb (II), Zn (II) and Ga (III) - only Cu (II), at concentrations higher than 10^−6^ M, and As (III) and Pb (II), at concentrations higher than 10^−4^ M, gave rise to peaks in the same range of potentials.

### 3.3. Analytical Applications

Leishmaniasis is an inflammatory disease, occurring in tropical regions, which affects 12 million people worldwide, and 1.5-2 million new cases of leishmaniasis are estimated to occur annually [35]. Treatment with antimonial drugs is the preferred method of fighting off this disease. The first generation of antimonial drugs contained Sb (III). However, despite its clinical benefits, because of its toxic side effects, a second generation of antimonial drugs was developed based on Sb (V).

The procedure described in this paper was applied to the determination of total antimony concentration in a commercial sample of Glucantime®. Reduction of Sb (V) to Sb (III) is necessary step prior to measurement. Substances that have been used for this purpose include hydrazine sulphate, sulphur dioxide and a combination of sodium disulphate and potassium iodide [36-40]. The use of L-cysteine to reduce and stabilize antimony in solution, as well as to decrease interference from transition metals and complex copper ions, has been reported [30, 41, 42]. In this work, the Sb (V) contained in the sample was reduced to Sb (III) with L-cysteine.

The analysis of the total concentration of Sb in the drug was made by standard addition. The analysis, completed in triplicate, obtained a total antimony concentration of 6.86 × 10^−1^ ± 0.34 × 10^−1^ M (n=3, α = 0.05). Good agreement was obtained between the concentration found and the values as supplied by the manufacturer (6.98 × 10^−1^ ± 0.35 × 10^−1^ M). These results were also checked using ICP-MS as a reference technique obtaining 6.90 × 10^−1^ ± 0.46 × 10^−1^ M (n = 3, α = 0.05) for total antimony concentration.

An analysis of Cantabrian Sea water near an industrial area, was performed using mercury film screen-printed electrodes. The reduction of Sb(V) to Sb(III) was also carried out as above was described. The total antimony concentration 1.58 × 10^−8^ ± 0.06 × 10^−8^ M (n = 3, α = 0.05) found by the proposed method and the values 1.62 × 10^−8^ ± 0.33 × 10^−8^ M (n = 3, α = 0.05) obtained using ICP-MS as reference technique show good agreement.

## 4. Experimental Section

### 4.1 Reagents and Apparatus

#### 4.1.1. Reagents

All solutions were prepared with deionised water obtained with a Barnstead NANO Pure II system. Stock standard solution of Sb (III) was obtained by dissolving potassium antimony tartrate (III) (analytical-reagent grade, Sigma, Steinheim, Germany) in water. A solution of Hg (II) 800 mg L^−1^ was prepared by dissolving the appropriate amount of mercury [28] chloride (analytical-reagent grade, Panreac, Barcelona, Spain) in 1.30 M hydrochloric acid (30 % suprapur grade, Merck, Darmstadt, Germany). Electrodag PF-407 A (carbon ink), Electrodag 418 SS (silver ink), Electrodag 6037 SS (silver/silverchloride ink) and Electrodag 452 SS (dielectric ink) were obtained from Achenson Colloiden (Scheemda, The Netherlands).

#### 4.1.2. Apparatus

SPEs were produced on a DEK 248 printing machine (DEK, Weymouth, UK) using polyester screens with appropriate stencil designs mounted at 45° to the printer stroke. Voltammetric measurements were taken using an Autolab PGSTAT 12 electrochemical system with GPES software (Eco Chemie, Utrecht, The Netherlands).

#### 4.1.3. Software

Data analysis was performed using the Statgraphics statistical software package [31] for the experimental design and Progress [32] for the robust regression.

### 4.2. Procedure

#### 4.2.1. Construction of SPEs

In this study, hand-made SPEs were used in the determination of Sb(III). A three-electrode configuration (working electrode, reference electrode and an auxiliary electrode) was constructed for the determination of Sb(III). Since, shape, surface area and spatial arrangement of the electrodes significantly influence the quality of the analytical response, an important stage in the construction of the electrode system is their design. In order to assemble the SPEs, successive layers of different inks were printed onto a PVC strip substrate (30 mm × 10 mm, 0.5 mm thick) using four different screens with an appropriate stencil in order to reach the required design. The design and printing procedure employed in this work has been described in previous works [43, 44]

#### 4.2.2. Mercury Film Preparation

In a separate process, the mercury film was coated over the screen-printed working electrode surface, using a solution containing 800 mg L^−1^of Hg (II). The deposition was performed by applying a potential of − 0.90 V for a period of time under stirring.

#### 4.2.3. Anodic Stripping Voltammetry Measurements

Voltammetric measurements were taken using the following procedure. In a solution of 3.00 M HCl containing the required antimony concentration a deposition potential of −0.70 V was applied for a period of time. The deposition step was performed under stirring. When that time had elapsed, the stirrer was subsequently switched off, and the solution was left to settle for 10 s, after which the voltammogram was recorded by making an anodic sweep from −0.70 V to 0.00 V, using a potential step of 0.006 V. The modulation time was 0.04 s and the interval time of the applied pulses was 0.60 s. In the cell solution a 8.00 mg L^−1^of Hg (II) concentration is always present in order to recondition the mercury film between each measurement [29, 30].

## Figures and Tables

**Table 1.** ANOVA with the data of the 2^3^ factorial design for optimization of experimental variables in Sb (III) determination by DPASV.

**Effect**	SS*	DF*	MS*	F_ratio_*	P_level_*
A: t_dep (Hg_	58.70	1	58.70	3.01	0.22
B: E_dep_	844.40	1	844.40	43.25	0.02 (a)
C: td_dep_	2118.68	1	2118.68	108.51	0.01 (a)
AB	313.12	1	313.12	16.04	0.06
AC	2.75	1	2.75	0.14	0.74
BC	280.49	1	280.49	14.37	0.06
Lack of fit	300.04	2	150.02	7.68	0.11
Pure error	39.05	2	19.52		
Total	3957.23	10			
R^2^ = 0.91					

* SS, sum of squares; DF, degrees of freedom; MS, mean squares; F _ratio_ : MS_factor_/M_Serror_; P _level_, probability level; (a) Significant factor at α = 0.05.

**Table 2.** ANOVA with the data of the 2^2^ central composite design for optimization of experimental variables in Sb (III) determination by DPASV.

**Effect**	SS*	DF*	MS*	F_ratio_*	P_level_*
A: E_dep_	1973.24	1	1973.24	92.76	0.01 (a)
B: t_dep_	1526.14	1	1526.14	71.74	0.01 (a)
AA	7142.17	1	7142.17	335.74	0.003 (a)
AB	110.25	1	110.25	5.18	0.15
BB	2096.10	1	2096.10	98.53	0.01 (a)
Lack of fit	1066.40	3	355.45	16.71	0.06
Pure error	42.55	2	21.27		
Total	12340.20	10			
R^2^ = 0.91					

* SS, sum of squares; DF, degrees of freedom; MS, mean squares; F _ratio_ : MS_factor_/MS_error_; P _level_, probability level; (a) Significant factor at α = 0.05.

**Table 3.** Calibration parameters obtained for electrode repeatability calculation.

	**1^st^ Calibration**	**2^nd^ Calibration**	**3^rd^ Calibration**
	
**Sensitivity × 10^−7^ (μA mol^−1^ M^−1^ dm^3^)**	3.34	3.59	3.55
**Intercept (μA)**	−6.67	−5.74	−4.49
**Residual Standard Deviation**	0.56	0.45	0.19
**Coefficient of determination. (R^2^)**	0.99	0.99	0.99

## References

[1] Furuta N., Iijima A., Kambe A., Sakai K., Sato K. (2005). Concentrations, enrichment and predominant sources of Sb and other trace elements in size classified airborne particulate matter collected in Tokyo from 1995 to 2004. J. Environ. Monit..

[2] Palchetti I., Cagnini A., Mascini M., Turner A.P.F. (1999). Characterisation of screen-printed electrodes for detection of heavy metals. Microchim. Acta.

[3] Quentel F., Filella M. (2002). Determination of inorganic antimony species in seawater by differential pulse anodic stripping voltammetry: stability of the trivalent state. Anal. Chim. Acta.

[4] Krachler M., Emons H., Zheng J. (2001). Speciation of antimony for the 21st century: promises and pitfalls. Trac-Trend Anal. Chem..

[5] Gillain G. (1982). Studies of pretreatments in the determination of Zn, Cd, Pb, Cu, Sb and Bi in suspended particulate matter and plankton by differential-pulse anodic-stripping voltammetry with a hanging mercury drop electrode. Talanta.

[6] Sturgeon R.E., Willie S.N., Berman S.S. (1985). Preconcentration of selenium and antimony from seawater for determination by graphite-furnace atomic-absorption spectrometry. Anal. Chem..

[7] Capodaglio G., Vandenberg C.M.G., Scarponi G. (1987). Determination of antimony in seawater by cathodic stripping voltammetry. J. Electroanal. Chem..

[8] Shotyk W., Krachler M., Chen B. (2006). Contamination of Canadian and European bottled waters with antimony from PET containers. J. Environ. Monit..

[9] Guo T.Z., Baasner J. (1993). Online microwave sample pretreatment for the determination of mercury in blood by flow-injection cold vapor atomic-absorption spectrometry. Talanta.

[10] Kadara R.O., Tothill I.E. (2004). Stripping chronopotentiometric measurements of lead(II) and cadmium(II) in soils extracts and wastewaters using a bismuth film screen-printed electrode assembly. Anal. Bioanal. Chem..

[11] Smichowski P., Madrid Y., Camara C. (1998). Analytical methods for antimony speciation in waters at trace and ultratrace levels. A review. Fresenius J. Anal. Chem..

[12] Wagner W., Sander S., Henze G. (1996). Trace analysis of antimony (III) and antimony (V) by adsorptive stripping voltammetry. Fresenius J. Anal. Chem..

[13] Bond A.M., Kratsis S., Newman O.M.G. (1998). Combined use of differential pulse adsorptive and anodic stripping techniques for the determination of antimony(III) and antimony(V) in zinc electrolyte. Anal. Chim. Acta.

[14] Wang J., Lu J.M., Tian B.M., Yarnitzky C. (1993). Screen-Printed Ultramicroelectrode Arrays for on-Site Stripping Measurements of Trace-Metals. J. Electroanal. Chem..

[15] Yarnitzky C., Wang J., Tian B.M. (2000). Hand-held lead analyzer. Talanta.

[16] Ugo P., Moretto L.M., Bertoncello P., Wang J. (1998). Determination of trace mercury in saltwaters at screen-printed electrodes modified with sumichelate Q10R. Electroanal.

[17] Desmond D., Lane B., Alderman J., Hill M., Arrigan D.W.M., Glennon J.D. (1998). An environmental monitoring system for trace metals using stripping voltammetry. Sensor Actuator B-Chem..

[18] Jasinski M., Grundler P., Flechsig G.U., Wang J. (2001). Anodic stripping voltammetry with a heated mercury film on a screen-printed carbon electrode. Electroanal..

[19] Honeychurch K.C., Hart J.P. (2003). Screen-printed electrochemical sensors for monitoring metal pollutants. Trac-Trends Anal. Chem..

[20] Beni V., Ogurtsov V.I., Bakunin N.V., Arrigan D.W.M., Hill M. (2005). Development of a portable electroanalytical system for the stripping voltammetry of metals: Determination of copper in acetic acid soil extracts. Anal. Chim. Acta.

[21] Palchetti H., Laschi S., Mascini M. (2005). Miniaturised stripping-based carbon modified sensor for in field analysis of heavy metals. Anal. Chim. Acta.

[22] Rodriguez B.B., Bolbot J.A., Tothill I.E. (2004). Urease-glutamic dehydrogenase biosensor for screening heavy metals in water and soil samples. Anal. Bioanal. Chem..

[23] Honeychurch K.C., Hawkins D.M., Hart J.P., Cowell D.C. (2002). Voltammetric behaviour and trace determination of copper at a mercury-free screen-printed carbon electrode. Talanta.

[24] Kadara R.O., Tothill L.E. (2005). Resolving the copper interference effect on the stripping chronopotentiometric response of lead(II) obtained at bismuth film screen-printed electrode. Talanta.

[25] Palchetti I., Majid S., Kicela A., Marrazza G., Mascini M. (2003). Polymer-mercury coated screen-printed sensors for electrochemical stripping analysis of heavy metals. Int. J. Environ. Anal. Chem..

[26] Zen J.M., Yang C.C., Kumar A.S. (2002). Voltammetric behavior and trace determination of Pb2+ at a mercury-free screen-printed silver electrode. Anal. Chim. Acta.

[27] Choi J.Y., Seo K., Cho S.R., Oh J.R., Kahng S.H., Park J. (2001). Screen-printed anodic stripping voltammetric sensor containing HgO for heavy metal analysis. Anal. Chim. Acta.

[28] Rajesh, Bisht V., Takashima W., Kaneto K. (2005). An amperometric urea biosensor based on covalent immobilization of urease onto an electrochemically prepared copolymer poly (N-3-aminopropyl pyrrole-co-pyrrole) film. Biomaterials.

[29] Adeloju S.B., Young T.M. (1995). Anodic-stripping potentiometric determination of antimony in environmental materials. Anal. Chim. Acta.

[30] Adeloju S.B., Young T.M., Jagner D., Batley G.E. (1998). Anodic stripping potentiometric determination of antimony on a combined electrode. Analyst.

[31] Statgraphics Centurion.

[32] Rousseeuw P.J., Leroy A. M. (1987). Robust Regression and Outlier Detection.

[33] Massart D.L., Vandeginste B.G.M., Buydens L.M.C., de Jong S., Lewi P.J., Smeyers-Verbeke J., Mann C.K. (1998). Handbook of Chemometrics and Qualimetrics, Part A.

[34] (2000). Capability of Detection. Part 2: Methodology in the Linear Calibration Case (11843-2).

[35] Flores E.M.D., da Silva F.E.B., dos Santos E.P., Paula F.R., Barin J.S., Zanella R., Dressler V.L., Bittencourt C.F. (2002). Determination of total arsenic by batch hydride generation atomic absorption spectrometry in injectable drugs containing high levels of Sb(V) as N-methylglucamine antimonate. Spectrochim. Acta B.

[36] Batley G.E., Florence T.M. (1974). Evaluation and comparison of some techniques of anodic-stripping voltammetry. J. Electroanal. Chem..

[37] Postupolski A., Golimowski J. (1991). Trace determination of antimony and bismuth in snow and water samples by stripping voltammetry. Electroanal..

[38] Gillain G., Rutagengwa J. (1985). Determination of Zn, Cd, Pb, Cu, Sb and bi in milk by differential pulse anodic-stripping voltammetry with a hanging mercury drop electrode following 2 independent mineralization methods. Analusis.

[39] Costantini S., Giordano R., Rizzica M., Benedetti F. (1985). Applicability of anodic-stripping voltammetry and graphite-furnace atomic-absorption spectrometry to the determination of antimony in biological matrices - A comparative-study. Analyst.

[40] Mok W.M., Wai C.M. (1987). Simultaneous extraction of trivalent and pentavalent antimony and arsenic species in natural-waters for neutron-activation analysis. Anal. Chem..

[41] Welz B., Sucmanova M. (1993). L-cysteine as a reducing and releasing agent for the determination of antimony and arsenic using flow-injection hydride generation atomic-absorption spectrometry. 1. Optimization of the analytical parameters. Analyst.

[42] Welz B., Sucmanova M. (1993). L-cysteine as a reducing and releasing agent for the determination of antimony and arsenic using flow-injection hydride generation atomic-absorption spectrometry .2. Interference studies and the analysis of copper and steel. Analyst.

[43] Domínguez-Renedo O., Arcos-Martínez M.J. (2007). Anodic stripping voltammetry of antimony using gold nanoparticle-modified carbon screen-printed electrodes. Anal. Chim. Acta.

[44] Domínguez-Renedo O., Arcos-Martínez M.J. (2007). A novel method for the anodic stripping voltammetry determination of Sb(III) using silver nanoparticle-modified screen-printed electrodes. Electrochem. Commun..

